# Establishing Reference Ranges of Hematological Parameters from Malian Healthy Adults

**DOI:** 10.4172/2165-7831.1000154

**Published:** 2017-03-15

**Authors:** B Kone, M Maiga, B Baya, YDS Sarro, N Coulibaly, A Kone, B Diarra, M Sanogo, ACG Togo, D Goita, M Dembele, MA Polis, J Warfield, M Belson, S Dao, S Orsega, RL Murphy, S Diallo, S Siddiqui

**Affiliations:** 1 University of Sciences, Techniques and Technologies of Bamako, Mali; 2 Leidos Biomedical Research, Inc., in support to NIAID, Bethesda, Maryland, USA; 3 Northwestern University, Chicago, Illinois, USA; 4 National Institute of Allergic and Infectious Diseases (NIAID), Bethesda, Maryland, USA

**Keywords:** Hematology, Adults, Mali

## Abstract

**Introduction:**

Measurement of immuno-hematological parameters has been historically helpful in the diagnosis and treatment monitoring of many infectious diseases and cancers. However, these parameters have not yet been established in many developing countries where patient care strongly relies on such low-cost tests. This study describes the immuno-hematological parameter ranges for Malian healthy adults.

**Methods:**

A cross sectional study was conducted from August 2004 to May 2013. We included 213 healthy volunteers (173 male and 40 female), aged between 18–59 years. Median, 2.5 and 97.5 percentile ranges for each immuno-hematological parameter are presented.

**Results:**

In our study population, the hematological parameters’ ranges were mostly different to the universal established ranges. We found in our population a Median white blood cell (WBC) count of 5200 cells/μL [3237.5–11900], Red Blood Cell (RBC) count of 4.94 10^6 [3.56–6.17], hemoglobin (Hb) of 14.2 g/dL [12.2–17.38], platelet count (Plt) of 275 10^3/μL [145.4–614.4], lymphocytes 2050/μL [1200–3800], neutrophils 2200/μL [1040–6220]; monocytes 200/μL [100–660]; eosinophils 131/μL [0–1026]; CD4 902 cells/μL [444–1669] and CD8 485 cells/μL [0–1272]. We found significant gender differences in RBC, Hb level and MPV. However, RBC and Hb were higher in males median values compared to females (median values) (p<0.001), whereas the Mean platelet volume lower values (MPV) in males than females (P<0.047). The hemoglobin level for some West African countries (Mali, Burkina Faso, Togo, and Nigeria) ranged from 13.5 to 15.1 g/dL for males and 12 to 13 g/dL for females. However in East and Southern Africa, the values were anywhere from 14.1 to 16.1 for males and 11.2 to 14.4 for females.

**Conclusion:**

Our data may help physicians to better define hematological abnormalities in patients. They may also be used to define new “normal hematological values” in Malian population or in the whole West African population.

## Introduction

Hematological and immunological parameter references ranges are widely used in clinical practice to assess health and disease conditions. The reference ranges could also be important tools as biomarkers to assess disease progression or response to therapy [1]. These parameters may vary depending on age, gender, race, environmental and genetic background [2]. Recent improvements in counting peripheral blood cells has given the field of hematology enormous clinical and scientific advantages [3–5], and more availability and access to such data to clinicians worldwide. The reference ranges universally used are mostly from studies conducted in Western countries even though they are sometimes different to those of other populations across the world. In Mali, studies determining hematological parameters have mostly been conducted in infants and children [6–7]. However no study has described the Immuno-hematological parameters in healthy adult subjects.

## Materials and Methods

### Study design, setting and population

A cross sectional study was conducted from August 2004 to May 2013. Participants were recruited at Point-G University Teaching Hospital and the laboratory testing was done at the SEREFO laboratory of the University of Sciences, Techniques and Technologies of Bamako (USTTB). The study population consisted of healthy volunteers aged 18 years or older, living in Bamako and willing to be part of the study. We defined a healthy volunteer, as a participant with no clinical evidence of TB or HIV and no physical symptom of illness for more than 2 weeks before enrollment, including no evidence of fever, or weight loss.

Two hundred and thirteen volunteers were enrolled consecutively after signing informed consent. Pre-screening tests included a physical examination and rapid hemoglobin testing using HemoCue Hb 201+ (USA/CANADA, FRIWO, Mod nr FE3515060D035) and pregnancy testing for women. Volunteers with a normal physical exam, a hemoglobin level more than 10 g/dL and women with a negative pregnancy test were enrolled in the study. This research protocol was approved by the Ethics Committee of the USTTB, Mali and the Institutional Review Board of the National Institute of Allergy and Infectious Diseases, National Institutes of Health, Bethesda. Maryland.

### Laboratory methods

#### HIV status determination

Whole blood was collected in 5 mL of serum separating tube (BD Vacutainer^®^ plus, Becton 17 K2EDTA, Becton Dickinson, Franklin Lakes, NJ, USA). HIV testing was performed using the following algorithm: A rapid test was done using Determine^®^ (HIV-1/2, Abbott Laboratories, Matsudo-Shi, Chiba, Japan) for all the participants, followed by an ELISA test (Genscreen Ag-Ac UltraHIV-1/2 version 2 Assay, Bio-Rad Laboratories, Marnes, France). Any positive ELISA was further confirmed by Western Blot testing (New Lav Blot I and II, Bio-Rad Laboratories, Marnes, France).

#### Hematological parameters testing

A coulter counter analyzer (Coulter Ac T diff, Beckman Coulter, Miami, FL) standardized by 4C plus control blood was used. The following hematological parameters were measured: White Blood Cell (WBC), Red Blood Cell (RBC), hemoglobin (Hb), hematocrit (Hct), Mean Cell Volume (MCV), Mean Cell Hemoglobin (MCH), mean Cell Hemoglobin Concentration (MCHC), Red Cell Distribution Width (RDW), platelet count (Plt), and Mean Platelet Volume (MPV). A Blood smear was also performed for manual WBC cell differentiation and determination of percentages and absolute values of Lymphocytes, Neutrophils, Eosinophils, Basophils and Monocytes for each participant.

All laboratory equipment used had passed Quality controlled assessment with Interlaboratory Quality Assurance Program (IQAP) and the College of American Pathologist (CAP-FH2) programs which is performed on a yearly basis since 2004.

#### Flow cytometry testing

CD4 and CD8 T Lymphocyte counts were performed using a FacsCalibur (FASCalibur, BD, Biosciences, San Jose, CA, USA). Whole blood was stained using a lyse/wash immunophenotyping procedure. One hundred microliter of peripheral whole blood was added into a polystyrene tube containing 20 μL of a monoclonal antibody cocktail of anti-CD3 Fluorescein isothiocyanate (FITC), anti-CD8 Phycoerythrin (PE), anti-CD45 Peridinin chlorophyll protein coupled to the cyanine dye Cy™ 5.5 (PercCp-Cy5.5) and anti-CD4 allophycocyanin (APC). Cells and antibodies were then incubated in dark for 15 min at room temperature. Thereafter, 2 mL of lysis buffer (BD Biosciences, San Jose, CA, USA) was added to the cells and kept for 10 min in dark at room temperature to allow red cell lyses.

Cells were then washed twice with 2 mL of BD Wash Buffer (Becton Dickinson) and re-suspended in 200 μL of BD Wash Buffer for acquisition with a four-color flow cytometer (FASCalibur, BD Biosciences, San Jose, CA, USA). Data were later analyzed with FlowJo software (Tree Star inc. Ashland, Oregon, USA).

#### Data and Statistical Analysis

Data were collected using study specific case report forms by a physician and upon validation was entered into an electronic clinical database (CRIMSON system (version 1.0)). Statistical analysis was performed using SPSS (version 16.0). The percentile range (2.5%–97.5%) was used to determine the higher and lower values of normal ranges.

## Results

Two hundred and fifty one (251) volunteers were screened during the study period, 15.1% (38/251) of whom were HIV positive and were therefore excluded from our analysis. Data from the remaining 213 volunteers were analyzed. Eighty one percent (173/213) were men and 19% (40/213) were women. The median age was 25.5 years [22–31.5].

The 2.5–97.5 percentile intervals and median values for each hematological parameters from the general population were mostly different to what comes with the instruments and usually used in routine laboratories in Mali (Table 1). The immuno-hematological parameters analysis by gender shows a median WBC count of 5200 cells per microliter with a range of (3075–11137) for men and 4700 cells/mL (3800–12500) for women but the difference was no statistically significant. Median Hemoglobin for males was 14.5 g/dL (12.4–17.6) and 12.8 g/dL (12.0–14.9) for females. For RBC, the median is 5.14 × 10^6/μL (4.16–6.23) for men and 4.67 × 10^6/μL (3.88–5.73) for women. The statistical difference for both parameters were significant (p = 0.001) (Table 1). The median MPV for males was lower than females’ MPV and this difference was significant. The lymphocytes median value was 2100 cells/μL for males and 2200 cells/μL for females. The range for hematological parameters according to gender and age ranges are described in Table 2. The neutrophil count ranged in males from 1000 to 4400 cells/μL and 1200 to 7400 cells/μL for females. The monocytes’ range was 100–660 cells/μL for males and 100–500 cells/μL for females. The eosinophils’ range was 0–1026 cells/μL for males and 0–3405 cells/μL for females. The CD4 and CD8 ranges in males were respectively [468–1636] cells/μl, [312–1272] cells/μl and for females [391–1748] cells/μl, and [364–1748] cells/μl (Table 1). There was no statistically significant gender difference in the median platelet counts.

## Discussion

Our data show differences with the reference data that come with the instruments and used in Mali routine laboratories. This study is the first to the best or our knowledge to determine hematological ranges in Malian adult population. However, because of limited sample size, our data cannot be extrapolated to the entire country. We found significant gender differences in RBC, Hb and MPV in the participants. RBC and Hb were higher in males than females (P< 0.001), and lower value MPV in males when compare to female (P< 0.047). These lower values are consistent with the differences usually seen globally and may be explained by menstrual blood loss [8]. The median value of hemoglobin for West African countries like Mali, Burkina Faso, Togo, and Nigeria ranged from 13.5 to 15.1 g/dL for males and 12 to 13 g/dL for females [9–11]. However for East and southern Africa these values were anywhere from 14.1 to 16.1 for males and 11.2 to 14.4 for the females (Table 3)”. These higher values for Eastern and Southern African populations when compared to West Africans are attributed to the impact of the high altitude in those regions leading to decreased oxygen in the air, and increased hematopoiesis in these populations [12].

In this study, we established the baseline immuno-hematological values in adult healthy volunteers in Malian population. Simple and easy to perform tests such as Blood Cell Counts are valuable in developing countries where lack of infrastructure and resources prevent more sophisticated and expensive tests. Physicians usually have to rely on symptoms alone to diagnose diseases. Current “normal values” are usually based on a Caucasian population and may not be applicable universally. Therefore it is important to determine the basic parameters for each genetically distinct population. It has to be noted that most of these parameters by themselves are not sufficient to diagnose diseases as they are not specific to a single disease. However, when combined with disease symptoms, epidemiological context and clinical signs, they could be very helpful to orient to diagnostic hypotheses.

In this study the median WBC absolute count was 5200 cells per microliter with a range of [3075–11137] for men and 4700 cells/mL [3800–12500] for women. There was no significant gender difference and our data are similar to other studies from Burkina Faso in West Africa and Ethiopia in East Africa [13]. However, these ranges are wider than that were found elsewhere in Africa [14]. Our ranges are different from the reference ranges used in Mali routine laboratories (Table 2). These data used by the routine laboratories are from the instruments’ manufacturer established from Western populations who are genetically, environmentally, socially and/or culturally different from African populations [15,16].

The hemoglobin ranges from this study were similar to Gambian values in men, but different in female [1]. We could explain this difference by our inclusion criteria of Hb level greater than 10 g/dL. Also the Eosinophil count in Mali ranged above the expected “normal” range in men and women. This is important as it may suggest exposure to allergens or hypersensitivity creating agents in the environment. It has to be noted that our inclusion criteria of Hb>10.5 may pick some borderline anemic subjects. Similarly, the females included were in the age of 18–49, and menstrual bleeding may influence the study outcomes. However, these hypotheses are less likely given the similarity of our data with previous findings in West Africa.

The CD4 and CD8 T-cell counts’ ranges in males were respectively [468–1636] cells/μL, [312–1272] cells/μL and for females [391–1748] cells/μL, [364–1748] cells/μL. The CD4 and CD8 T-cells counts ranges were similar to Uganda, Tanzanian, and Mozambique reports [2,14,17]. Some studies in Africa have shown that the median platelet count is significantly higher in females than males [17]. However, we did not find any statistically significant gender difference in our study population regarding platelets values.

It has to be noted that lower and higher ranges in this study is more meaningful than the statistically significance in the differences.

## Conclusion

This study was carried out to describes the local hematological and immunological parameters within Malian adults healthy volunteers, and our data have shown different parameter ranges, which can be utilized by physicians to help clinicians investigate diseases. We also demonstrated that the universally defined “normal values” are not always applicable to our Malian population.

## Figures and Tables

**Table 1:** Gender differences in immuno-hematological parameters: median, 2.5 and 97.5 percentile reference range.

Parameters n	Male		Female	
	Lab Value (n=173)	Range for routine Lab	Lab Value (n=40)	Range for routine Lab
WBC (/μL)	5200 (3075–11137)	(4000–10000)	4700 (3800–12500)	(4000–10000)
RBC (× 10^6 /μL)	5.14 (4.16–6.23)	(4.50–5.50)	4.67 (3.88–5.75)	(4.20–5.20)
Hb (g/dL)	14.5 (12.4–17.6)	(13,0–16,0)	12.8 (12.0–14.9)	(11,5–15,0)
Hct (%)	43.5 (33.2–54.6)	(40.0–54.0)	39.5 (26.8–52.5)	(37.0–47.0)
MCV (fL)	87.3 (72.3–97.7)	(75–95)	86.0 (39.2–118.0)	(75.0–95.0)
MCH (pg)	28.6 (22.8–33.7)	(30–35)	28.4 (23.1–34.8)	(30–35)
MCHC (g/dL)	33.0(30.9–34.9)	(30–35)	32.5 (30.9–34.5)	(30–35)
RDW (%)	13.4 (11.9–17.1)	(10–15)	13.7 (11.6–24.3)	(10–15)
Plt (× 10^3/μL)	259 (133–460)	(150–500)	291 (151–532)	(150–500)
MPV (fL)	7.8 (5.7–9.4)	(6.5–11)	8.0 (6.0–10.5)	(6.5–11)
Neutrophils (/μL)	2200 (1000–4400)	(2500–7500)	2200 (1200–7400)	(2500–7500)
Lymphocytes (/μL)	2100 (1200–3800)	(1000–4000)	2200 (1400–4600)	(1000–4000)
Monocytes (/μL)	200 (100–660)	(400–1000)	200 (100–500)	(400–1000)
Eosinophils (/μL)	165 (0–1026)	(40–500)	74 (0–825)	(40–500)
Basophils (/μL)	37 (0–108)	(inf-100)	0 (0–108)	(inf-100)
CD4 Count (cells/μL)	915 (468–1636)	N/A	902 (391–1748)	N/A
CD8 Count (cells/μL)	483 (312–1272)	N/A	494 (364–1748)	N/A

**Table 2:** Immuno-hematological parameters of 2.5 and 97.5 percentile reference range according to age and gender.

Parameter	Men age (Years)	n	Range values	Female age (years)	n	Range values

WBC (/μL)	18–49	163	2972–8802	18–49	40	3800–12500
	50–59	11	3900–26500			
RBC (× 10^6 /μL)	18–49	163	4.15–6.24	18–49	40	3.88–5.75
	50–59	11	4.24–6.14			
Hb (g/dL)	18–49	163	12.4–17.5	18–49	40	12.0–14.9
	50–59	11	14.0–17.9			
HCT (%)	18–49	163	33.1–54.6	18–49	40	26.8–52.5
	50–59	11	37.2–54.6			
MCV(fL)	18–49	163	72.1–96.4	18–49	40	39.2–118.0
	50–59	11	72.7–101.1			
MCH (pg)	18–49	163	23.1–32.9	18–49	40	23.1–34.8
	50–59	11	22.8–35.1			
MCHC (g/dL)	18–49	163	30.9–34.7	18–49	40	30.9–34.5
	50–59	11	31.4–36.0			
RDW (%)	18–49	163	11.9–17.4	18–49	40	11.6–24.3
	50–59	11	12.3–16.3			
PLT (× 10^3)	18–49	163	142–482	18–49	40	151–532
	50–59	11	117–332			
MPV (FL)	18–49	163	5.6–9.3	18–49	40	6.0–10.5
	50–59	11	6.1–9.8			
Neutrophils (× 10^2 /μL)	18–49	163	1000–4462	18–49	40	1200–7400
	50–59	11	1800–2400			
Lymphocytes (× 10^2 /μL)	18–49	163	1200–3800	18–49	40	1400–4600
	50–59	11	1800–2800			
Monocytes (× 10^2/μL)	18–49	163	100–672	18–49	40	100–500
	50–59	11	0–400			
Eosinophils (× 10^2/μL)	18–49	163	0–1006	18–49	40	0–825
	50–59	11	0–1508			
Basophils (× 10^2 μL)	18–49	163	0–110	18–49	40	0–108
	50–59	11	0–100			
CD4 Counts (× 10^2 cells/μL)	18–49	163	468–1656	18–49	40	391–1748
	50–59	11	480–999			
CD8 Counts (× 10^2 cells/p)	18–49	163	0–1263.6	18–49	40	364–1748
	50–59	11	0–1272.0			

**Table 3:** Immuno-hematological parameters’ comparison with other studies in Africa.

Study	WBC (10^9/liter)		RBC 10^12/liter		Hemoglobin (g/dl)		Neutrophil (%)		Platelet(10^9/liter)		Lymphocyte (%)	
	Male	Female	Male	Female	Male	Female	Male	Female	Male	Female	Male	Female
Our Study	5.2	4.7	5.1	4.7	14.5	12.8	44	48	259	291	2.1*	2.2*
N=213	(3.07–11.1)	(3.8–12.5)	(4.1–6.2)	(3.9–5.7)	(12.4–17.6)	(12.0–14.9)	(26–66)	(26–67)	(133–460)	(151–460)	(1.2–3.8)	(1.2–7.4)
(18–59) years												
Burkina (7)												
N=186	5.1	5.2	4.7	4.2	13.5	12	43	46	217	252	2.1	2.2*
(18–78 years	(3.2–9.2)	(3.4–7.4)	(11.3–15.6)	(3.5–4.9)	(11.3–15.6	(9.813.5)	(27–64)	(30–62)	(127–365)	(159–356)	(1.3–4.0)*	(1.4–3.2)
Togo (8)	4.1	4.2	5	4.5	15.1	13	1.6*	1.6*	236	247	2.1*	2.2*
N=1379	(1.9–10.1)	(2.2–7.8)	(3.3–6.4)	(3.1–6.0)	(10–18.4)	(10.3–17.1)	(0.5–5.4)	(0.5–4.4)	(120–443)	(150–436)	(1.1–4.3)	(1.2–4.3)
(15–58) years												
Nigeria (9)	4.4	4.5	5.2	4.56	14.3	12.8	53	51	213	236	40	41
N=383	(4.3–4.6)	(4.4–4.8)	(5.1–5.3)	(5.5–5.3)	(14.0–14.4)	(12.4–13.1)	(52.6–55.2)	(49.1–52.3)	(206.8–226.8)	(229.3–251.2)	(37.4–40.2)	(39.0–42.1)
(18–65) years												
Ethiopia (11) N=485**	5.9	5.9	5	4.5	16.1	14.4			203	193	1801*	1701*
(15–45) years	(3.0 ± 9.8)	(3.0 ± 12.2)	(4.3 ± 5.9)	(3.7 ± 5.2)	(13.9 ± 18.3)	(12.2± 16.6)	NA	NA	(97 ± 324)	(98 ± 352)	(956 ± 3474)	(1098 ± 3487)
Mozambique (15)	4.6	5.6	5.1	4.2	14.1	11.2	52.5	57.1	231.1	269	1.8*	1.9*
N=257	(2.9–7.7)	(3.2–9.1)	(2.7–6.1)	(2.3–5.0)	(12.3–16.4)	(7.0–13.1)	(34.4–70.8)	(37.0–76.7)	(116.2–392.1)	(128.8–503)	(1.1–3.3)	(1.0–3.1)
(18–24) years												
Tanzania (12)	4.4	4.8	5.2	4.6	15.4	13.5	47.5	48.4	224	271	1.8*	1.9*
N=301	(2.8–7.9)	(3.2–8.0)	(4.4–6.3)	(3.8–5.5)	(13.7–17.7)	(11.1–15.7)	(31.7–69.3)	(32.5–71.3)	(147–356)	(151–425)	(1.1–2.8)	(1.1–3.1)
(19–48) years												
Uganda (2)			4.9	4.5	14.1	12.5			171	198		
N=845	NA	NA	(3.8–6.0)	(3.7–5.3)	(11.1–16.8)	(10.1–14.3)	NA	NA	(80–288)	(100–297)	NA	NA
>24 years												
